# Supplementary material for: Saccharomyces boulardii (CNCM I-745) improves intestinal damage in sepsis by remodeling intestinal flora structure
Source: Front Cell Infect Microbiol. 2025 Jul 31;15:1602792. doi: 10.3389/fcimb.2025.1602792 (PMC12350329; doi:10.3389/fcimb.2025.1602792)
Supplement: Supplementary file 1 [file DataSheet1.zip › summary/summary/7_Differential_analysis/2_LEfSe_result/Group_DW4_vs_DW0_vs_CW4_vs_CW0_vs_BW4_vs_BW0_vs_AW4_vs_AW0_0.05_3.0/LEfSe_Group_DW4_vs_DW0_vs_CW4_vs_CW0_vs_BW4_vs_BW0_vs_AW4_vs_AW0.abund.pdf]

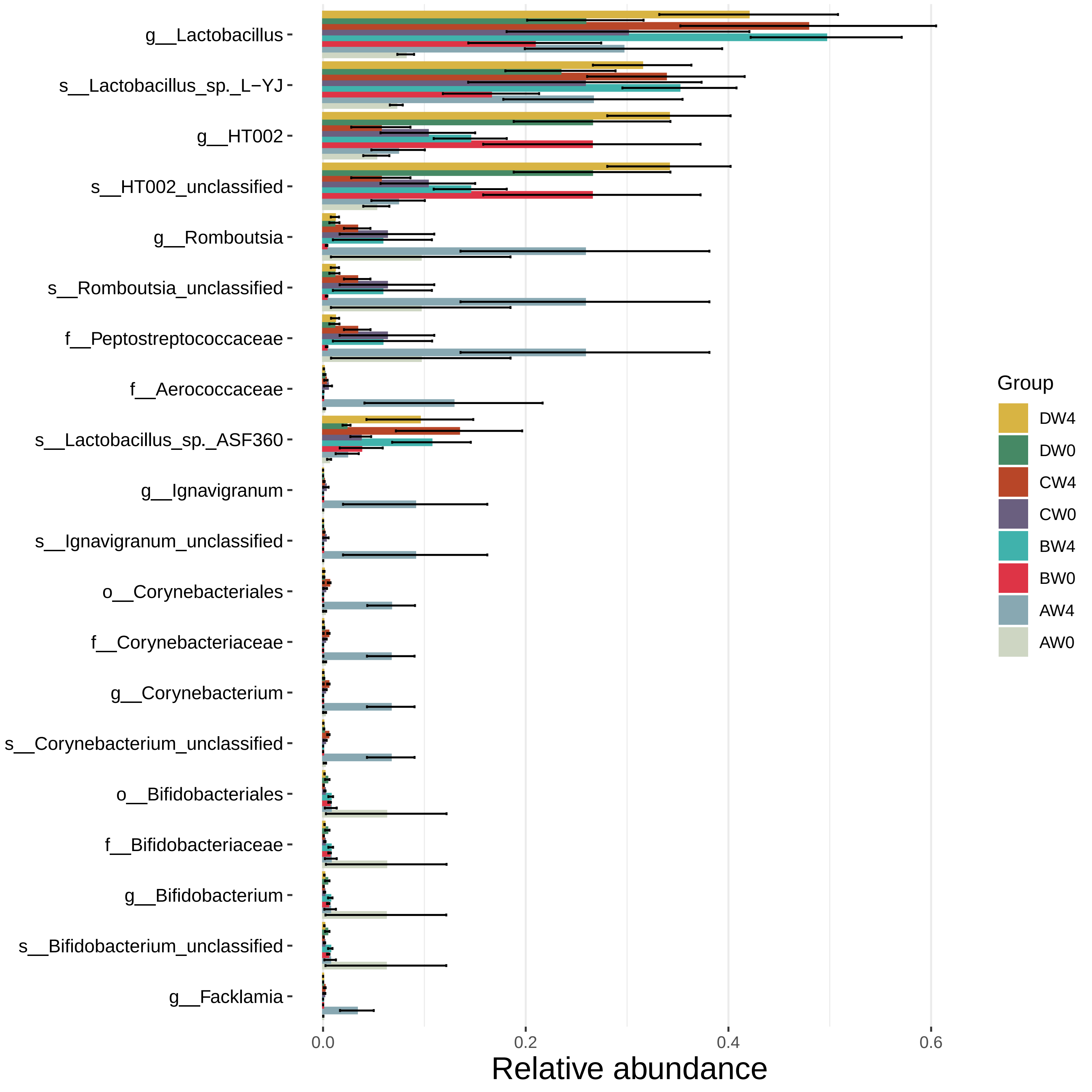

g__Lactobacillus
s__Lactobacillus_sp._L−YJ
g__HT002
s__HT002_unclassified
g__Romboutsia
s__Romboutsia_unclassified
f__Peptostreptococcaceae
f__Aerococcaceae
s__Lactobacillus_sp._ASF360
g__Ignavigranum
s__Ignavigranum_unclassified
o__Corynebacteriales
f__Corynebacteriaceae
g__Corynebacterium
s__Corynebacterium_unclassified
o__Bifidobacteriales
f__Bifidobacteriaceae
g__Bifidobacterium
s__Bifidobacterium_unclassified
g__Facklamia
0.0
0.2
0.4
0.6
Relative abundance
Group
DW4
DW0
CW4
CW0
BW4
BW0
AW4
AW0
